# Analgesic effect of α-terpineol on neuropathic pain induced by chronic constriction injury in rat sciatic nerve: Involvement of spinal microglial cells and inflammatory cytokines

**DOI:** 10.22038/IJBMS.2019.14028

**Published:** 2019-12

**Authors:** Mohsen Soleimani, Mohammad Abbas Sheikholeslami, Shiva Ghafghazi, Ramin Pouriran, Siavash Parvardeh

**Affiliations:** 1 Department of Pharmacology, School of Medicine, Shahid Beheshti University of Medical Sciences, Tehran, Iran

**Keywords:** Alpha-terpineol, Inflammatory cytokines, Microglial cells, Neuropathic pain, Rat, Sciatic nerve

## Abstract

**Objective(s):**

Neuropathic pain is a prevalent and debilitating neurological disorder. Ample evidence indicates that microglial cells and inflammatory cytokines are involved in the pathogenesis of neuropathic pain. Alpha-terpineol is a monoterpenoid alcohol with inhibitory effect on inflammatory cytokines. The main purpose of this study was to evaluate the effect of α-terpineol on neuropathic pain in rats.

**Materials and Methods:**

Chronic constriction injury (CCI) model was utilized to induce neuropathic pain in male *Wistar* rats. The rats were randomly divided into control, sham, α-terpineol, and gabapentin groups. Normal saline, α-terpineol (25, 50, and 100 mg/kg), and gabapentin (100 mg/kg) were administered intraperitoneally in the above-mentioned groups once daily for 14 days post-CCI. Behavioral tests, including Von Frey, acetone, and Hargreaves were used to assess mechanical allodynia, cold allodynia, and hyperalgesia in rats. Iba1 immunostaining and ELISA procedures were used to assess the activation of microglial cells and inflammatory cytokines level.

**Results:**

The results showed that α-terpineol (50 and 100 mg/kg) significantly attenuated mechanical allodynia, cold allodynia, and hyperalgesia in the neuropathic rats. The analgesic effect of α-terpineol (100 mg/kg) was comparable with that of gabapentin as a standard antineuropathic pain drug. In addition, α-terpineol (25, 50 and 100 mg/kg) significantly decreased the number of Iba1-positive cells and diminished the concentration of IL-1β and TNF-α in the spinal tissue.

**Conclusion:**

It was ultimately attained that α-terpineol attenuates neuropathic pain through the suppression of the microglial cells and reduction of inflammatory cytokine levels in the spinal cord of rats.

## Introduction

Neuropathic pain is a debilitating neurological disease which occurs basically as a consequence of disruption of neurons’ structure and function, particularly in the somatosensory system (1). Neuropathic pain frequently is observed in patients with diabetic neuropathy, multiple sclerosis, spinal cord injury, postherpetic neuralgia, and cancer, which imposes severe restrictions on the normal activities of life due to its chronicity and poor outcomes (2). A great deal of attempt has been made to manage neuropathic pain; however, the currently used medications have not shown a desirable efficacy (3). Hence, the efforts are still ongoing to create new medications for the management of neuropathic pain.

There are several mechanisms underlying the pathogenesis of neuropathic pain (2, 4, 5). The currently available evidence shows that inflammatory cytokines, such as tumor necrosis factor alpha (TNF-α), interleukin-1 beta (IL-1β), and IL-6, which are secreted by immune cells contribute to the induction and progression of peripheral neuropathy (6, 7). Although under normal conditions, the inflammatory cytokines are expressed in low amounts in the neural tissues, their concentrations are increased obviously after nerve injury. In fact, regardless of various underlying factors and diversity in symptoms, elevated levels of inflammatory cytokines are considered as the most indispensable common feature of different kinds of neuropathic pains. Many studies have revealed that glial cell activation plays a pivotal role in overexpressing and releasing the excessive amounts of inflammatory cytokines during the nerve injury, which in turn increases the excitability of neurons and potentiating the transduction of pain signals from the periphery to the central nervous system (6, 8). The overactivity of glial cells, particularly in the spinal cord will ultimately result in experiencing severe pain in the absence of noxious stimuli (allodynia) or excess in hypersensitivity states in response to noxious stimuli (hyperalgesia) (6). Accordingly, it seems reasonable, that suppressing the glial cells could alleviate neuropathic pain (6, 8). Apparently, those molecules which suppress glial cells activation or at least inhibit the release of inflammatory cytokines significantly can alleviate neuropathic pain effectively (9).

Recently, researchers have used medicinal plants in the management of neuropathic pain. In this regard, the beneficial effects of the extracts and essential oils of various medicinal herbs have been reported in the animal models of neuropathic pain (10, 11). However, in the majority of these studies, the pharmacological effects of total extract or essential oils have been explored, and it is not clear which of the constituents in these plants may be the underlying cause of their analgesic effects. 

Alpha-terpineol, a natural monoterpenoid alcohol, is known as the major constituent in the essential oils in some medicinal plants such as *Eucalyptus* spp (12). Several pharmacological effects have been known for α-terpineol, including anticonvulsant (13) and neuroprotective (14, 15). In addition, α-terpineol represented a reduction effect on compound action potential (CAP) in rat sciatic nerve, which indicated an inhibitory effect on voltage-dependent sodium channels (16). Besides, α-terpineol possesses antinociceptive effect in the formalin test and remarkably decreases pain response, especially in the second phase of the formalin test (17, 18). There is evidence that indicates the involvement of inflammatory mechanisms in the second phase of formalin test (19). Consistent with these reports, α-terpineol has shown a significant ability to attenuate hyperalgesia which is induced by TNF-α (20). Furthermore, α-terpineol inhibits lipopolysaccharide-induced hyperexpression of inflammatory cytokines including TNF-α, IL-1β, and IL-6; while enhancing the anti-inflammatory cytokines, for instance IL-10 in macrophages (21). Considering the pharmacological effects of α-terpineol including antinociceptive and anti-inflammatory properties, as well as its inhibitory effect on the CAP conduction in rat sciatic nerve, we hypothesized that α-terpineol might be effective in attenuating neuropathic pain. Accordingly, the main purpose of this study was to evaluate the analgesic effects of α-terpineol on neuropathic pain by utilizing the chronic constriction injury model in rat sciatic nerve, and also exploring the underlying mechanism with a focus on microglial cells and inflammatory cytokines in the spinal cord. To the best of our knowledge this is the first report regarding the analgesic effect of α-terpineol in neuropathic pain.

## Materials and Methods

***Chemicals***

The chemicals and drugs used in this research were as follows: α-terpineol (Sigma Co., USA; CAS Number 98-55-5, PubChem Substance ID 329747989); gabapentin (Sigma), ketamine (Alfasan), and xylazine (Alfasan). The solutions were prepared freshly by using normal saline as a solvent. Alpha-terpineol was suspended in normal saline by using tween 80 (0.5% v/v). The mixture of normal saline and tween 80 was used as the vehicle.

***Animals***

Male *Wistar* rats weighing 200 to 250 g were used in this study. The animals were kept in standard cages under 12 hr light/dark cycle, the temperature was approximately 25±2 ^°^C, and the optimum humidity was met. The rats had free access to food and tap water except in times when they were under the experiment. All the procedures of this study were performed according to the EU Directive 2010/63/EU for animal experiments. The procedures were approved by the ethical committee of Shahid Beheshti University of Medical Sciences (# IR.SBMU.MSP.REC.1395.245). 

***Induction of neuropathic pain in rats***

In this study, the chronic constriction injury (CCI) method was utilized in order to induce neuropathic pain in rats. For this purpose, the rats’ sciatic nerve in the left paw was exposed under anesthesia with ketamine/xylazine (50/5 mg/kg, IP) and four chromic catgut ligatures were loosely tied over the nerve trunk. The ligatures were located just proximal to the trifurcation of the sciatic nerve. Then, the muscles and skin were sutured. Eventually, the animals were brought to the animal room for recovery (22). 

***Animal grouping and drug administration schedule***

The rats were divided into six groups (n=8 in each group) including control, sham, gabapentin (100 mg/kg, IP); and three α-terpineol-treated groups (25, 50, and 100 mg/kg, IP). In the control and sham groups, the animals received normal saline and tween 80 as the vehicle. In the sham-operated group, the rats were anesthetized, and their sciatic nerve was exposed; however, it was not ligated. The vehicle, gabapentin, and α-terpineol were administered once a day for 14 days post-CCI. The dosage of α-terpineol was selected based on previous studies (20, 23). 

***Behavioral tests for evaluation of neuropathic pain in rats***

Mechanical allodynia, cold allodynia, and hyperalgesia were assessed before the surgery (day 0) and also on days 3, 5, 7, 10, and 14 after sciatic nerve ligation.

**Figure 1:**
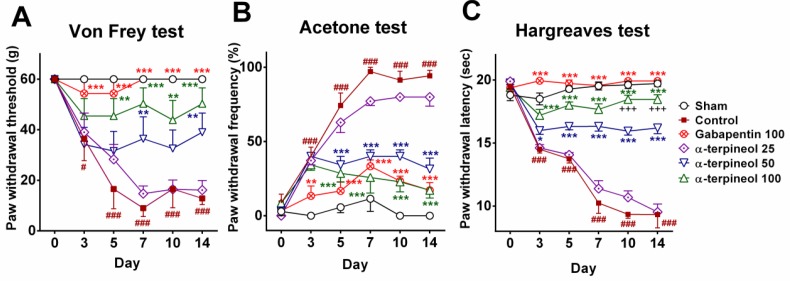
The effect of α-terpineol on CCI-induced neuropathic pain. Mechanical allodynia (A), cold allodynia (B), and thermal hyperalgesia (C) were evaluated on days 0 (before surgery) and 3, 5, 7, 10, and 14 following sciatic nerve ligation. Data are expressed as mean±SEM for 8 rats. *P< 0.05, **P<0.01, ***P<0.001 (compared to control); #*P*<0.05, ###*P*<0.001 (compared to sham); +++*P*<0.001 (compared to α-terpineol 50 mg/kg); CCI: Chronic constriction injury

**Figure 2:**
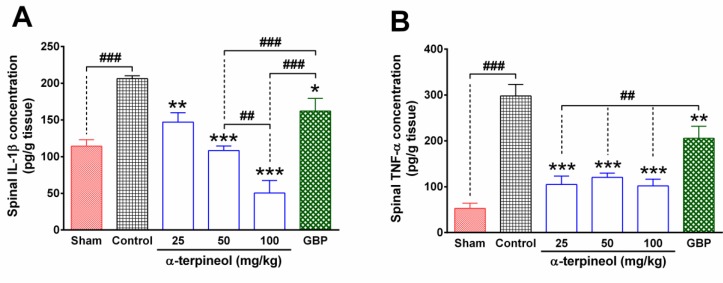
The effect of α-terpineol on spinal levels of inflammatory cytokines in rats subjected to neuropathic pain. The concentration of IL-1β and TNF-α were measured in the lumbar part of the spinal cord using the ELISA method on the 14th day after the ligation of the sciatic nerve. Samples were from four animals and were duplicated. Each column represents mean±SEM for four rats. *P<0.05, **P<0.01, ***P<0.001 (compared to control); ##*P*<0.05, ###*P*<0.001; GBP: gabapentin; Control: normal saline + tween 80

**Figure 3:**
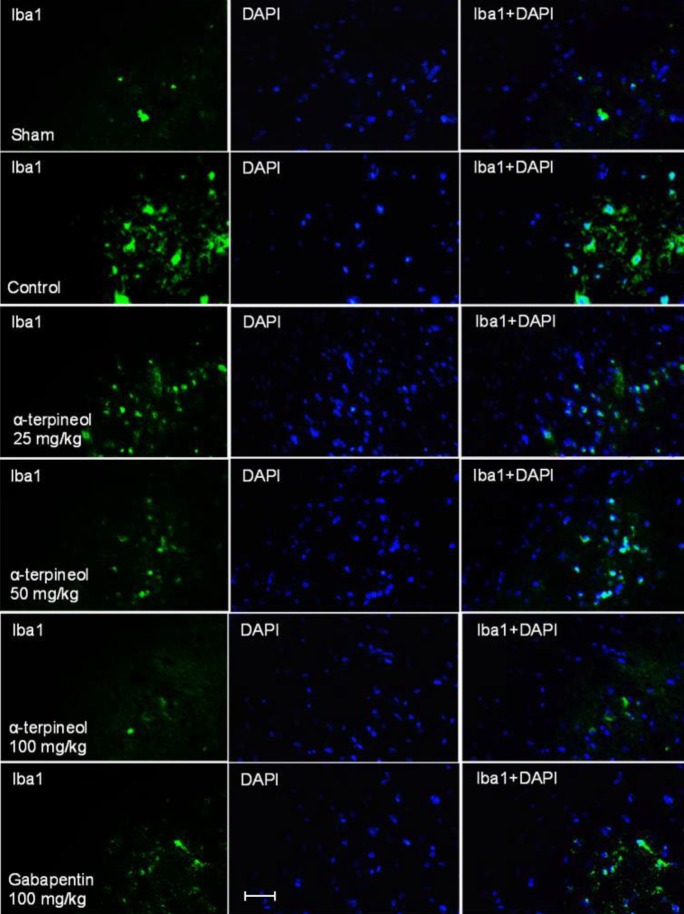
The effect of α-terpineol on spinal microglial activation in the rats subjected to neuropathic pain. Photomicrographs show immunofluorescence staining of ionized calcium-binding adapter molecule 1 (Iba1, green) in the lumbar part of spinal cord in different groups on the 14^th^ day after the ligation of sciatic nerve. DAPI (4′,6-diamidino-2-phenylindole) staining (blue) was used to identify cell nuclei. Magnification ×400; Scale bar: 20 micrometers; Control: normal saline + tween

**Figure 4:**
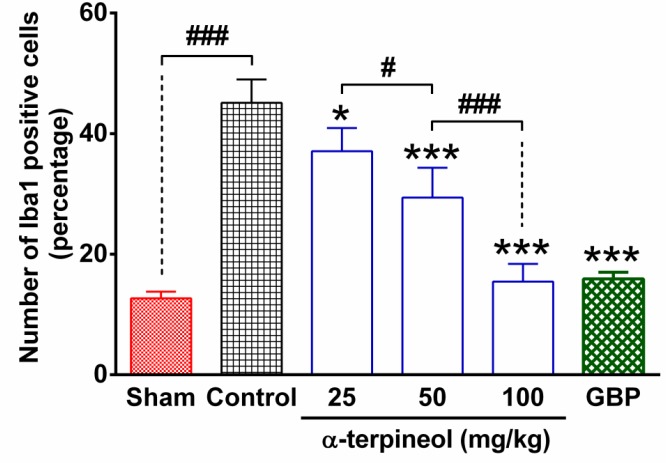
The number of Iba1 positive cells in the lumbar part of the spinal cord in rats that were subjected to neuropathic pain. Each column represents the percentage of ionized calcium-binding adapter molecule 1 (Iba1)-positive cells in the sham, control, α-terpineol-, and gabapentin-treated groups. Data from five slices of each sample were averaged and expressed as mean±SEM for each group (n=4). *P<0.05, ***P<0.001 (compared to control); #*P*<0.05, ###*P*<0.001; GBP: gabapentin; Control: normal saline + tween 80

***Mechanical allodynia***

Mechanical allodynia was evaluated in order to determine the level of sensitivity to touch as a non-noxious stimulation. The Von Frey test was performed by using a series of calibrated nylon filaments (Stoelting Co., USA). The filaments were applied in the order of 0.6, 1, 1.4, 2, 4, 6, 8, 10, 15, 26, and 60 g. Each filament was slightly touched at the right angle to the plantar surface of the injured paws for five times. The intervals were 10 sec, and the paw withdrawal threshold (PWT) was measured. The minimum gram strength eliciting three sequential pain responses was considered as PWT (22).

***Acetone-induced cold allodynia***

The acetone test was utilized to evaluate cold allodynia. In order to perform this procedure, one acetone drop (50 μl) was touched smoothly to the plantar surface of the injured paw and the animal reaction as paw withdrawing, shaking or licking was recorded as a pain response. The procedure was performed for each rat 5 times with the intervals of one min, and the percentage of paw withdrawal frequency (PWF) was calculated as follows: (number of paw withdrawals/5 trials)×100 (22).

***Thermal hyperalgesia***

In order to assess the α-terpineol effect on hyperalgesia, the Hargreaves test was used. In this test, a noxious heat stimulus which was produced by an infra-red light source (Plantar Analgesymeter, Harvard, USA) was shone directly beneath the injured paw, and paw withdrawal latency (PWL) was recorded. The procedure was carried out five times with the intervals of 5 min in each rat. The noxious heat stimulus stopped automatically in order to prevent tissue damage if the animal did not show any reaction within 20 min (22). 

***Measuring levels of inflammatory cytokines in the spinal cord***

At the end of the behavioral tests (day 14), four rats from each group were sacrificed, and the lumbar part of the spinal cord (segments L1–L6) was dissected out. The tissue levels of inflammatory cytokines including IL-1β and TNF-α were measured by using the sandwich enzyme-linked immunosorbent assay (ELISA) method (24). In order to perform this experiment, the spinal samples and standard solutions were prepared in accordance with the manufacturer’s instructions (Abcam). Briefly, the samples were first homogenized and lysed by using cell lysis buffer. Then, the lysates were centrifuged at 13000 rpm, 4^ °^C for 20 min. After that, the supernatants were transferred to new tubes and diluted 5-fold in sample diluent buffer. One hundred microliters of each sample and standard solution were then added to each well of rat IL-1β or TNF-α ELISA kits (Abcam, ab-100767 and ab-100785, UK). Biotinylated TNF-α or IL-1β antibodies, HRP-streptavidin, and TMB one step substrate were added to each well. Finally, stop solution was added to the wells and the absorbance of each well was read at 450 nm using an ELISA reader (Bioteck^®^, USA). The standard curve was used to determine the concentration of IL-1β and TNF-α in spinal samples. First, the optical density (OD) values of the standard were plotted against the known concentration of the standard solutions, and a concentration-OD scatter plot was obtained by using GraphPad Prism Software (Version 7). Then, the OD values of spinal samples were keyed into the software, and the concentration of IL-1β and TNF-α was extrapolated from the standard curve.

***Spinal tissue preparation for Iba1 immunostaining***

On the 14^th^ day of trial, four other animals from each group were perfused transcardially with normal saline (0.9%) and fixed with paraformaldehyde (4%). After removing lumbar part of fixed spinal cord, they were placed in tissue molds and were embedded in paraffin. Then, the spinal tissues were cut coronally into 5-micrometers slices and were placed on microscope’s slides and thus were prepared for immunostaining. In order to assess the spinal microglial activation, immunostaining of the ionized calcium-binding adapter molecule 1 (Iba1) was carried out by utilizing Anti-Iba1 antibody in accordance with the manufacturer’s instructions (Abcam). 

Briefly, the spinal samples were washed twice for 5 min each time in TBS (tris-buffered saline) and 0.025% Triton X-100 with gentle agitation. The samples were then incubated in 10% normal saline with 1% bovine serum albumin in TBS for 2 hr at room temperature. After incubation, the slides were drained for 5 seconds and then wiped around carefully with tissue paper. In the next step, the slides were incubated with primary antibody overnight at 4 ^°^C. The slides were rinsed twice for 5 min each time with TBS plus 0.025% Triton X-100 and then incubated in 0.3% H_2_O_2_ in TBS for 15 min. After incubation, the samples were exposed to secondary antibody for an hour at room temperature (25). At the end of staining, 4′,6-diamidino-2-phenylindole (DAPI) was used to identify the neurons’ nuclei. The slides were placed in DAPI for 20 min in room temperature and eventually washed with water for 5 min (26). Finally, the spinal microglial activation was examined under a fluorescence microscope at 400 × magnification. In addition, image analysis was carried out to quantify immunofluorescent staining of Iba1 in the spinal samples by using the ImageJ Software. 

***Statistical analysis***

The data was subjected for the analysis of variance (ANOVA) through one-way ANOVA, followed by mean comparisons by Tukey’s test, which was expressed as mean±SEM (standard error of the mean) for each group. One-way ANOVA with repeated measures was utilized to analyze behavioral tests on different days. Differences between means were considered significant if *P*<0.05.

## Results

***Effect of α-terpineol on mechanical allodynia***

Three days after the induction of neuropathic pain, mechanical allodynia was significantly increased in the control group (*P*<0.05), which persisted until the end of the experiment (*P*<0.001). In contrast, the administration of α-terpineol with the dosage of 50 mg/kg significantly increased PWT in rats, in the 7^th^ and 14^th^ days (*P*<0.01). The maximum dose of α-terpineol (100 mg/kg) caused a significant increase in PWT in rats from the 5^th^ to 14^th^ day (*P*<0.01). The treatment with gabapentin as the standard drug significantly increased the PWT on the 3^rd^ day, which persisted until the end of the experiment (*P*<0.001; Figure 1A). 

***Effect of α-terpineol on cold allodynia***

A significant increase in PWF was observed in the control group from the 3^rd^ to 14^th^ day (*P*<0.001). The administration of α-terpineol (50 and 100 mg/kg) decreased the PWF significantly in rats from the 5^th^ to 14^th^ day (*P*<0.001). Besides, gabapentin decreased the PWF significantly from the 3^rd^ to 14^th^ day. The analgesic effect of α-terpineol (100 mg/kg) was equal to the gabapentin on the 10^th^ and 14^th^ days (Figure 1B). 

***Effect of α-terpineol on hyperalgesia***

The results of Hargreaves test showed that 3 days after the constriction of sciatic nerve, the PWL was significantly decreased in the control group (*P*<0.001); while α-terpineol (50 and 100 mg/kg) significantly prolonged PWL in the neuropathic rats (*P*<0.001). The antihyperalgesic effect of α-terpineol with the dosage of 100 mg/kg was significantly greater than the dosage of 50 mg/kg on the 10^th^ and 14^th^ days (*P*<0.001). In addition, the administration of gabapentin increased the PWL significantly (*P*<0.001; Figure 1C). 

***Effect of α-terpineol on spinal levels of inflammatory cytokines***

The results of the ELISA test showed that the concentration of IL-1β and TNF-α in the lumbar part of the spinal cord significantly increased, following chronic constriction of the sciatic nerve (*P*<0.001). In contrast, α-terpineol (25, 50, and 100 mg/kg) significantly decreased the levels of IL-1β and TNF-α in the tissue (*P* <0.001; Figure 2). The higher dose of α-terpineol (100 mg/kg) resulted in a greater reduction in the spinal levels of IL-1β, compared to the dosage of 50 mg/kg (*P* <0.01). In addition, gabapentin significantly decreased the concentration of IL-1β (*P*<0.05) and TNF-α (*P*< 0.01) in the spinal tissues of rats that were subjected to neuropathic pain. The treatment of rats with α-terpineol resulted in more reduction in the spinal levels of IL-1β (*P*<0.001) and TNF-α (*P*<0.01), compared to the gabapentin group (Figure 2). 

***Effect of*** ***α*** ***-*** ***terpineol*** ***on microglial activation in the spinal cord***

According to the immunofluorescence analysis of fixed rat spinal tissue, which was done by the Iba1 antibody, it was attained the chronic constriction of sciatic nerve increased the intensity of immunostaining in the lumbar part of the spinal cord in the control group (Figure 3).

The statistical analysis indicated that the number of Iba1 positive cells in the lumbar part of the spinal cord was significantly higher in rats which were subjected to neuropathic pain, compared to the sham group (Figure 4). In contrast, the administration of α-terpineol (25, 50 and 100 mg/kg), reduced the intensity of immunostaining (Figure 3) and decreased dose-dependently the number of Iba1-positive cells in the spinal tissue, compared to the control group (Figure 4).

## Discussion

The clinical neuropathic pain is a debilitating and prevalent syndrome which arises from damage to the peripheral nervous system. Such damage results in both hyperalgesia and allodynia. Ample evidence from studies in rodents and humans has demonstrated the implication of microglial cells and inflammatory cytokines in peripheral neuropathic pain (6-8). Accordingly, it seems that those molecules which modulate the immune system can attenuate neuropathic pain effectively (9).

In the current study, the analgesic effect of α-terpineol on neuropathic pain was investigated by using the CCI model in rats. The obtained results demonstrated for the first time that α-terpineol attenuates remarkably hyperalgesia and allodynia in the rats that were subjected to neuropathic pain. Our findings clarified that the analgesic effect of α-terpineol is mediated through suppressing spinal microglial cells and reducing tissue levels of inflammatory cytokines. The attenuating effect of α-terpineol on neuropathic pain was exerted in a dose-dependent manner; therefore, by the increase in α-terpineol dosage, further reduction was observed in the antiallodynic and antihyperalgesic action of α-terpineol. 

The CCI model is one of the most commonly used experimental methods for simulation of neuropathic pain in rats. It apparently evokes both allodynia and hyperalgesia as the major manifestations of neuropathic pain (27). Moreover, the CCI method elicits the inflammatory processes mainly through activating glial cells in the injured sciatic nerve, followed by progression toward the spinal cord and disruption of the function of projection neurons that convey sensory information to higher centers (8, 27). 

In the peripheral neuropathies, microglial cells are dramatically activated, particularly in the dorsal horn where injured primary sensory neurons make synaptic connection with interneurons and projection neurons (28). Many studies have shown that during the peripheral neuropathic pain, immunostaining level of Iba1, as a microglia/macrophage-specific marker, is elevated in the spinal cord tissue and reaches the peak point within one to two weeks (7, 8). Consistent with these reports, our findings showed that constriction of rat sciatic nerve during two weeks led to a significant increase in the number of Iba1-immunopositive cells. In contrast, α-terpineol diminished the number of Iba1-positive cells in a dose-dependent manner and reduced the intensity of staining in the spinal cord. It was clearly compatible with its antiallodynic and antihyperalgesic activities in the behavioral tests. Besides, the result of ELISA test showed that the spinal levels of IL-1β and TNF-α in the control rats that were subjected to neuropathic pain increased significantly, which was compatible with the elevated immunostaining level of microglial cells. These findings are consistent with the previous studies showing that during the peripheral neuropathy, the inflammatory cytokines are produced largely and secreted by microglial cells. Currently available evidence shows that the released cytokines increase the glutamatergic synaptic transmission in the dorsal horn neurons and provoke the primary afferent neurons to release excitatory amino acids and substance P. These mechanisms explain the contribution of inflammatory cytokines in the neuropathic pain clearly (29). 

In the present study, the administration of α-terpineol resulted in a prominent decrease in spinal levels of IL-1β and TNF-α in rats. Our observations are consistent with the recent evidence indicating that systemic administration of α-terpineol attenuates the mechanical hypernociception in rat’s paw, which was induced by intraplantar injection of TNF-α (20). In addition, it has been shown that α-terpineol decreases the expression of TNF-α, IL-1β, and IL-6 in peritoneal macrophages that were induced by lipopolysaccharide (21). These reports reinforce the notion that α-terpineol might act as a potent anti-inflammatory agent. Moreover, it could ameliorate neuroinflammation occurring in different pathological circumstances. 

The present study also clarified that the suppressive effect of α-terpineol on spinal levels of IL-1β and TNF-α is more than that of gabapentin as the positive control. Gabapentin is an antiepileptic drug which functions through the inhibition of calcium currents underlying vesicular exocytosis and thereby causes a significant decline in neuronal activity (9). Recent studies demonstrated that gabapentin suppresses microglial activation and reduces tissue levels of inflammatory cytokines in the spinal cord subjected to neuropathic pain (30, 31). 

The present study not only supports these findings but also showed that α-terpineol could additionally suppress the activated microglia and further reduce the spinal levels of inflammatory cytokines, compared to the gabapentin. Nevertheless, the antihyperalgesic and antiallodynic effects of gabapentin in the behavioral tests were partially better than those of α-terpineol; however, the antiallodynic effect of α-terpineol in acetone tests equaled gabapentin in the 2^nd^ week of treatment. 

In line with the attained data, it was concluded that the attenuating effects of α-terpineol on the microglial activation and secretion of inflammatory cytokines in the spinal cord underlie its antihyperalgesic and antiallodynic effects. However, other possible mechanisms of action should not be ruled out completely. For instance, Moreira *et al*. showed that α-terpineol decreased the amplitude of evoked CAP and reduced the conduction velocity of the sciatic nerve in rats (16). This evidence suggests that the analgesic effect of α-terpineol on the neuropathic pain may be due to inhibitory effects on the nerve conduction partly through the blockade of voltage-dependent sodium channels. Besides, recently, it has been shown that α-terpineol exerts an anti-nociceptive effect through activating the serotonin receptors in a non-inflammatory chronic muscle pain model in rats (23). There is evidence indicating that some serotonin receptors including 5-hydroxytryptamine (5-HT) 2A and 5-HT3 in the spinal cord are responsible for antihyperalgesic and antiallodynic effects of serotonin receptor agonists in various animal models of neuropathic pain (32, 33). 

On the other hand, the involvement of nitric oxide (NO)/cyclic guanosine monophosphate (cGMP)/adenosine triphosphate-dependent potassium channel (K_ATP_) pathway has been demonstrated in antinociceptive and antineuropathic effects of different analgesic agents including gabapentin (34, 35). In this regard, Safaripour *et al*. showed that the signaling pathway of NO/cGMP/K_ATP_ channels plays an important role in the antinociceptive effect of α-terpineol in mice (18). Accordingly, there is a possibility that the NO/cGMP/K_ATP_ pathway plays an indispensable role in antiallodynic and antihyperalgesic effect of α-terpineol in the rat model of peripheral neuropathic pain. However, this effect deserves further investigations.

## Conclusion

The results of this study demonstrated that α-terpineol exerts analgesic effects in neuropathic pain that was induced by chronic constriction of rat sciatic nerve. In addition, the attained results indicated that the α-terpineol effect was exerted through the suppressing microglial cells and the reduction of inflammatory cytokines. Future works are needed regarding other mechanisms of analgesic effect of α-terpineol in neuropathic pain. 

## Conflicts of Interest

The authors declare that they have no conflicts of interests to disclose.
